# A Quality Improvement Project to Improve Experiences of Audio Quality for Remote Attendees of a Ward Round at a London Acute Adult Mixed Psychiatric Inpatient Ward

**DOI:** 10.1192/bjo.2022.320

**Published:** 2022-06-20

**Authors:** Hamilton Morrin, Vinitha Soundararajan, Rupa Ramesh, James Woollard

**Affiliations:** 1 Oxleas NHS Foundation Trust, London, United Kingdom; 2 King's College London, London, United Kingdom

## Abstract

**Aims:**

We sought to assess the degree, nature and impact of poor audio quality during ward rounds for individuals attending remotely using Microsoft Teams. We aimed to evaluate attendee experiences of audio quality against our expectation that due to the existing ward microphone system using a cardioid polar pickup pattern that attendees would have difficulty hearing all members of the multidisciplinary team, as well as the patient, gathered in the conference room. We also hypothesised that after switching to an omnidirectional sound recording system, we would observe an improvement in attendee satisfaction with audio quality during ward rounds.

**Methods:**

Individuals who had remotely attended ward rounds at Lesney Ward, Oxleas NHS Trust between 01/11/21 and 01/01/22, mainly patient family members and community care co-ordinators, completed a digital feedback questionnaire regarding audio quality. There was no exclusion criteria. Data from Likert scale questions were analysed with descriptive statistical tests (mode and distribution of responses). As minimal demographic data were obtained, inferential statistical tests were not used. Qualitative data were analysed using thematic sorting based on prevalence of themes in the data.

**Results:**

Feedback was provided by three family members, one ward team member and six members of the community mental health team prior to the intervention. Pre-intervention feedback indicated high levels of dissatisfaction with 6/10 respondents reporting they were “dissatisfied” and 1/10 “very dissatisfied”. Only 3/10 of attendees reported being able to hear and understand all individuals physically present in the room. In addition, respondents agreed that the audio quality was poor (modal response “bad”, 6/10), and that the sound quality impacted upon their experience of the ward round (modal response “yes, greatly”, 6/10).

The three most common main issues reported by respondents were: people speaking too far from the microphone (7/10), voices sounding muffled (6/10), and too much background noise (4/10). Using their own words, respondents described how the ward round sound quality made them feel. Common themes identified through thematic sorting included: distress, difficulty in understanding information / management plan, ward round prolongation and inability to comprehend the patient or staff.

**Conclusion:**

In conclusion, we found that when using an in-built laptop microphone with unidirectional pick-up remote ward round attendee satisfaction was poor, though this improved with the introduction of an omnidirectional system. Key areas for improvement include assessment of optimal positioning for adequate audio pick-up, and the introduction of automatic transcription for individuals with hearing impairments.

